# Results of Acute Treatment of Reverse Hill-Sachs Lesion Associated With Locked Posterior Shoulder Dislocation With Modified McLaughlin Procedure: A Case Report and Literature Review

**DOI:** 10.7759/cureus.77849

**Published:** 2025-01-22

**Authors:** Muhammed Kazez, Ali Sami Seker

**Affiliations:** 1 Orthopaedics and Traumatology, Elazığ Fethi Sekin Şehir Hastanesi, Elazığ, TUR

**Keywords:** acute treatment, locked posterior shoulder dislocation, modified mclaughlin procedure, reverse hill-sachs lesion, shoulder stability

## Abstract

This case report evaluates the clinical outcomes of two patients with locked posterior shoulder dislocation associated with a reverse Hill-Sachs lesion who were operated on in the acute phase with a modified McLaughlin procedure. Both patients had dislocated posterior shoulder fractures affecting approximately 40% of the articular surface of the humeral head and could not be reduced in the emergency department. Both patients were operated on the same day of hospitalization. We analyzed the clinical and radiological results after at least three years of follow-up. Care should be taken not to overlook these rare injuries and difficult reduction maneuvers should be avoided in the emergency department. Reduction and articular surface reconstruction with the modified McLaughlin method can be performed in the same session and in the acute period. Acute treatment of the reverse Hill-Sachs lesion associated with locked posterior shoulder dislocation with the modified McLaughlin procedure is effective and safe. Further studies are needed to determine the optimal treatment of these very rare injuries.

## Introduction

Posterior dislocation of the shoulder is probably one of the most common overlooked injuries and occurs approximately 25 times less frequently than anterior shoulder dislocations [1]. Posterior shoulder dislocation is more common after epileptic seizures and electric shocks [2]. Approximately half of posterior shoulder dislocations may be accompanied by an impaction fracture anteromedial to the humeral head called a reverse Hill-Sachs lesion, which can make shoulder reduction difficult because the glenoid is anterior to this locked area [3].

An overlooked or untreated posterior dislocation may result in permanent instability, osteonecrosis, chondrolysis and eventually arthrosis of the shoulder joint [4]. The most important parameters determining the treatment are the duration of the dislocation and the size of the humeral head defect. Humeral head defects of less than 25% can usually be treated with reduction and conservative measures, while defects greater than 50% usually require autograft or allograft transplantation, hemiarthroplasty or total shoulder arthroplasty [5]. When the defect is between 25% and 50% of the humeral head articular surface, surgical options have been described, including transfer of the subscapularis tendon (McLaughlin procedure) [6], block transfer of the subscapularis tendon and small tuberosity (modified McLaughlin) [6], rotational osteotomy [7], and autograft or allograft reconstruction [8].

In this study, we aimed to evaluate the clinical outcomes of two patients who underwent acute surgery with the modified McLaughlin procedure for the reverse Hill-Sachs lesion associated with locked posterior shoulder dislocation.

## Case presentation

**Figure 1:**
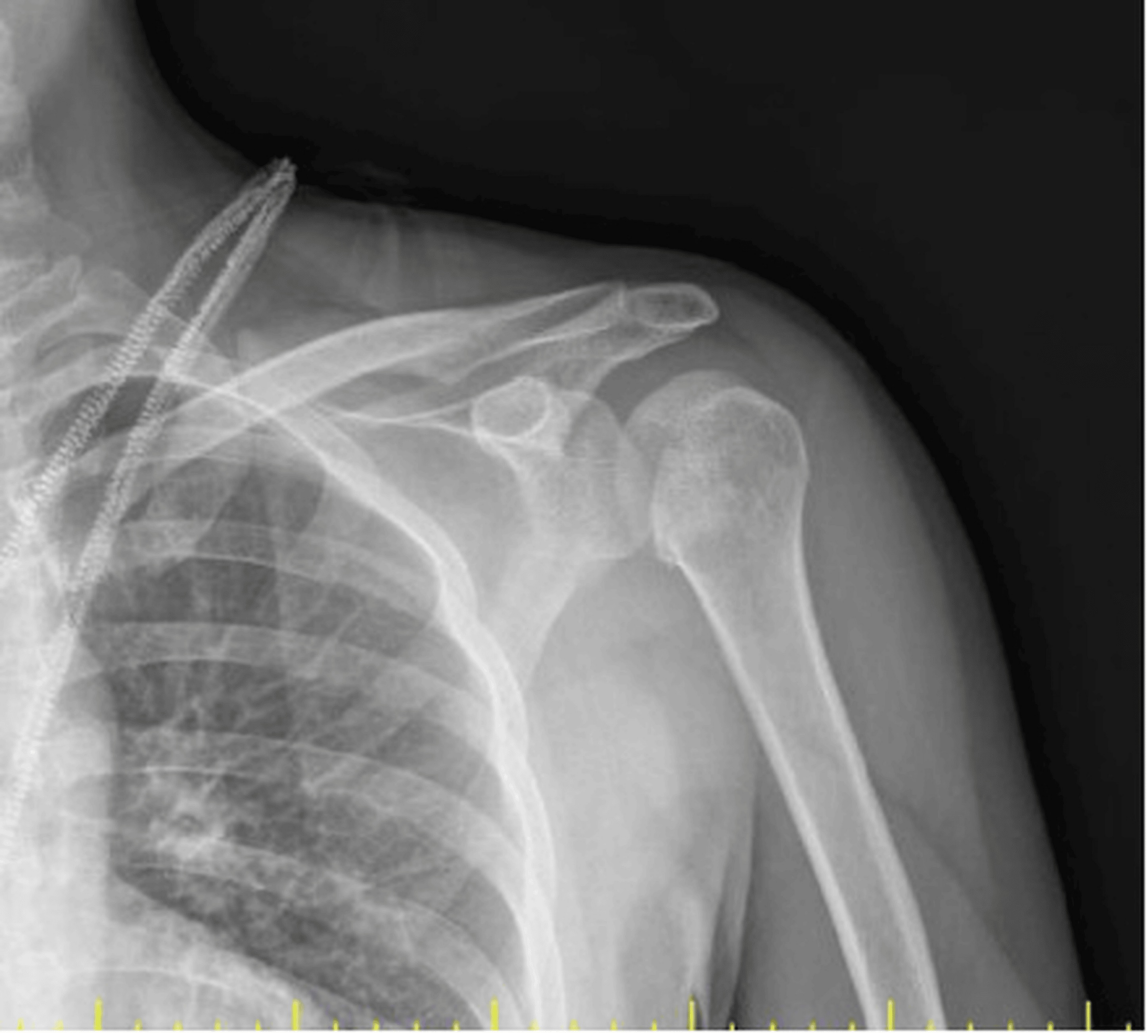
Left shoulder anterioposterior X-ray radiograph taken in the emergency department (Patient 1)

**Figure 2:**
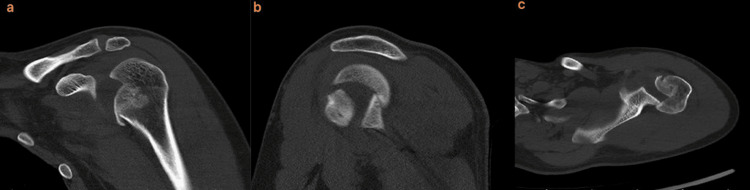
Left shoulder CT coronal (a), sagittal (b) and axial (c) sections taken in the emergency department (Patient 1)

The first patient was a 32-year-old male dental technician who presented with a left shoulder dislocation five days after a collision and fall on a carpet pitch. The second patient, a 30-year-old man, presented with a fall from a height while working in a construction site. Both patients had no additional injuries other than the humeral head defect accompanying posterior left shoulder dislocation (Figure 1). Both patients had no known comorbidity and no previous history of shoulder dislocation. Both patients were evaluated in the emergency department for shoulder dislocation and their dislocations could not be reduced under sedation anesthesia in the emergency department. Computed tomography (CT) of the two patients diagnosed with posterior locked shoulder dislocation showed a reverse Hill-Sachs lesion involving 35-40% of the joint anteromedial to the humeral head (Figure 2). Both patients were operated on the day of presentation. 

Surgical technique

**Figure 3:**
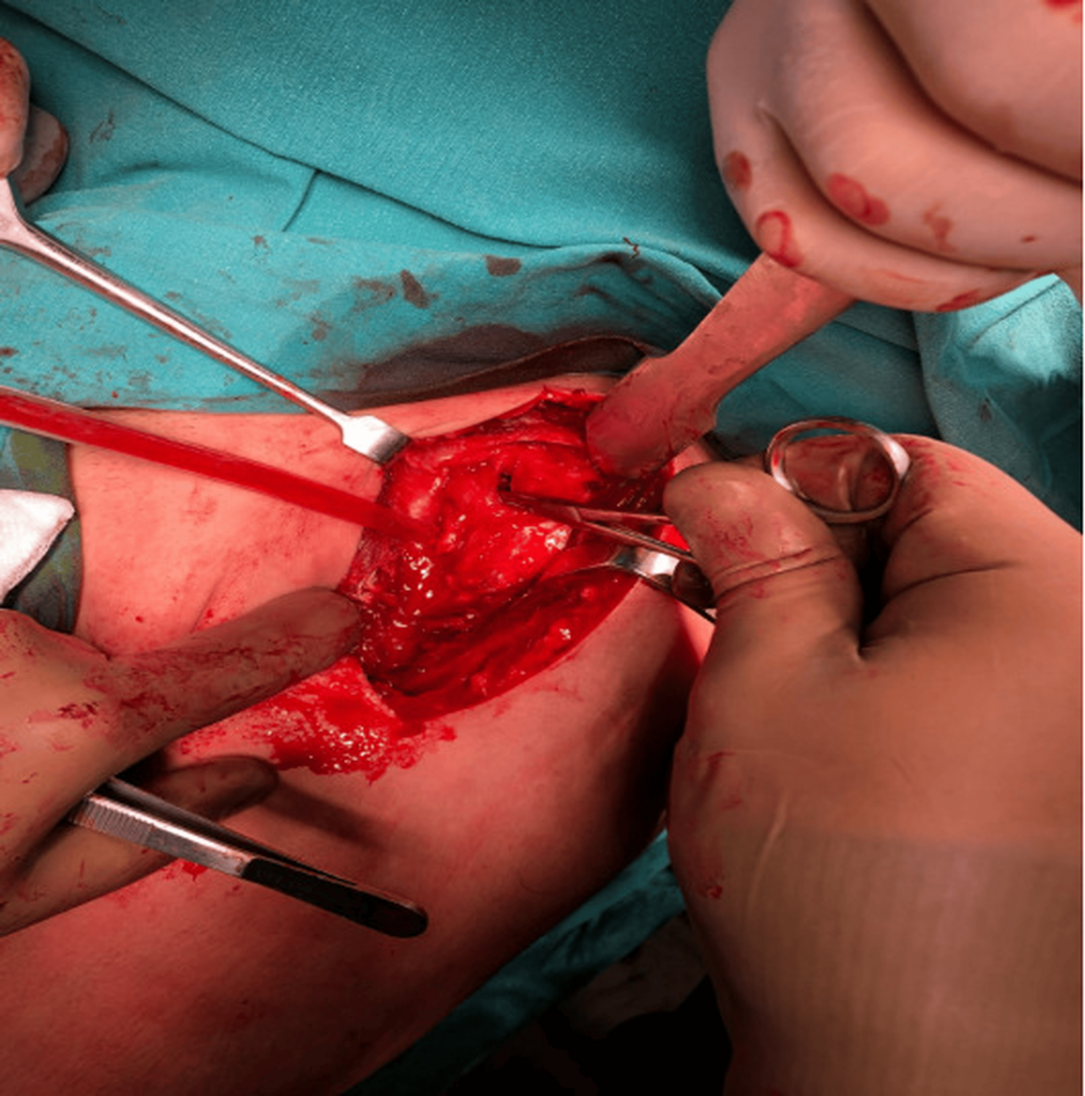
A blunt-tipped retractor was inserted through the rotator interval and the posterior locked shoulder dislocation was gently reduced to avoid further cartilage loss (Left shoulder, intraoperative image of Patient 1)

**Figure 4:**
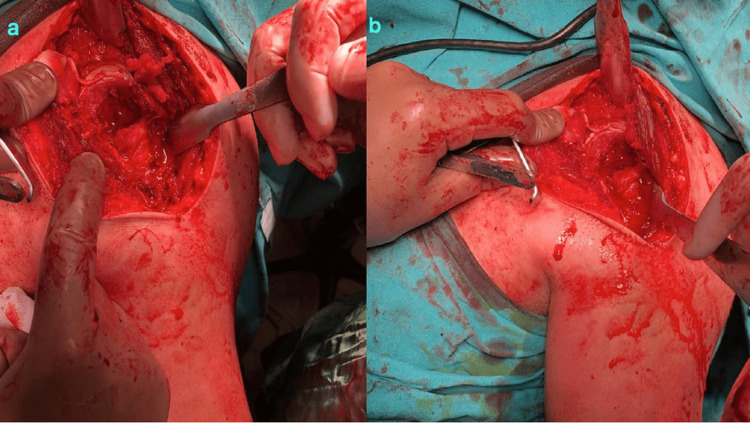
The impacted area in the joint was approximately 40% of the humeral head; (a) Patient 1 and (b) Patient 2

**Figure 5:**
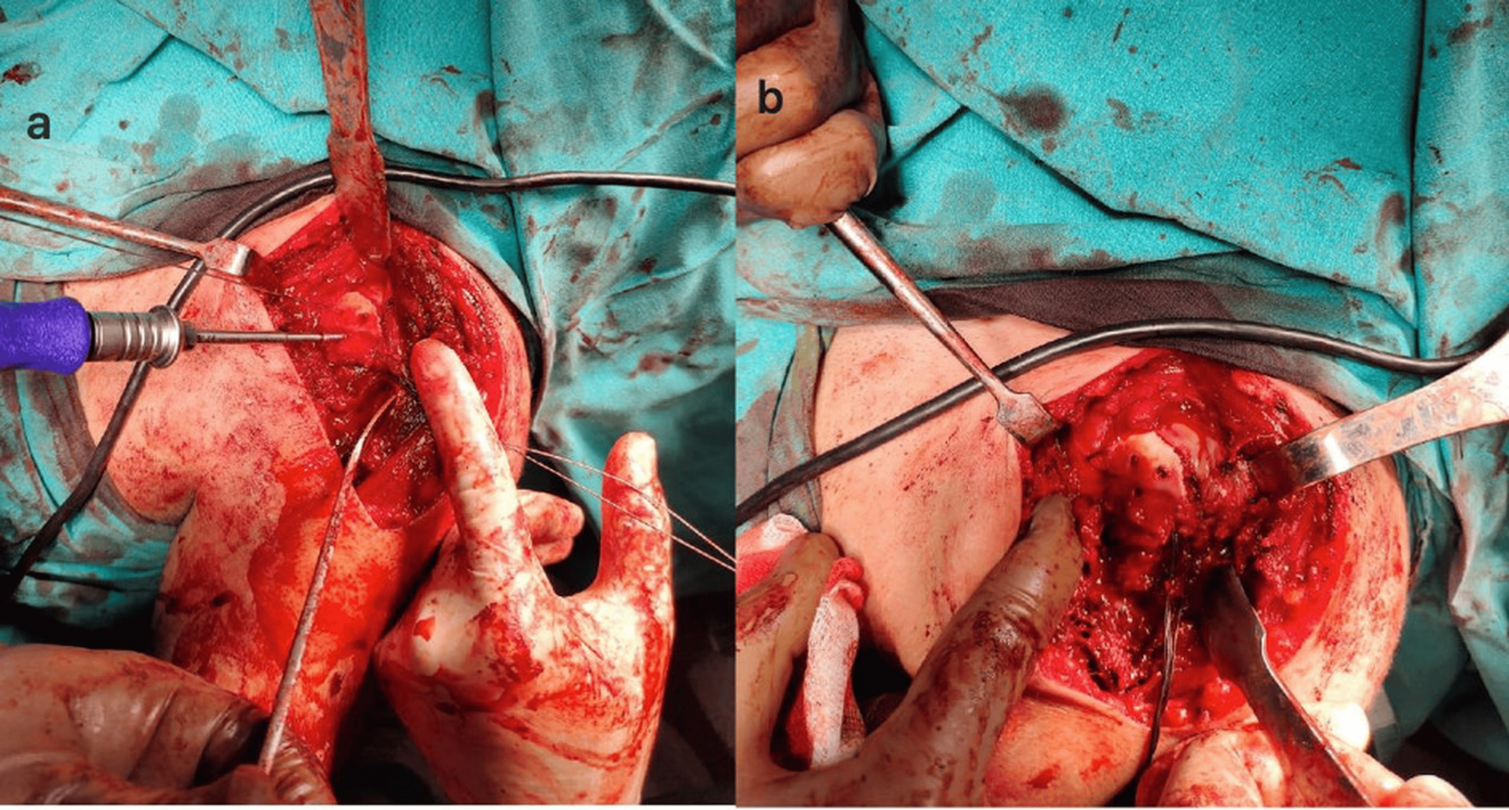
Intraoperative images of both patients who were fixed with cannulated screws after closure of the humeral head defect with tuberculum minus and reduction of the impacted articular cartilage. (a) Patient 1 and (b) Patient 2

**Figure 6:**
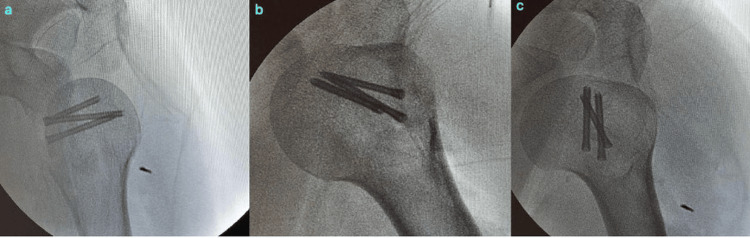
Intraoperative scopic images of Patient 1 in internal rotation (a), neutral (b) and external rotation (c) positions for anatomical reduction of the impacted fracture of the left humeral head and fixation with three cannulated screws

The operations were performed in the beach chair position. The deltopectoral approach was used. Firstly, the shoulder dislocations were gently reduced by entering through the rotator interval (Figure 3), then the impacted cartilage fragment in the humeral head was removed. In both patients, the humeral head defect was approximately 40% of the humeral head (Figure 4). Two holes were made in the tuberculum minus with K-wire (Kirschner wire) and reinforced sutures were passed through the holes, and then osteotomy was performed on the tuberculum minus without disturbing the biceps groove. The tuberculum minus was removed together with the subscapularis tendon and placed appropriately into the impaction defect in the humeral head. The sutures passed through the tuberculum minus were sutured transosseously to the humeral head. The previously removed piece of impacted articular cartilage was placed on the tuberculum minus in a way to ensure joint congruence and not to create a step. After a smooth articular surface was obtained, the articular cartilage was fixed with three cannulated screws (Figure 5). Anatomical reduction and control of fixation materials were confirmed by intraoperative scopic imaging (Figure 6). Tuberculum minus and subscapularis transfer (modified McLaughlin) was performed by the same surgeons in both patients.

Follow-up period

**Figure 7:**
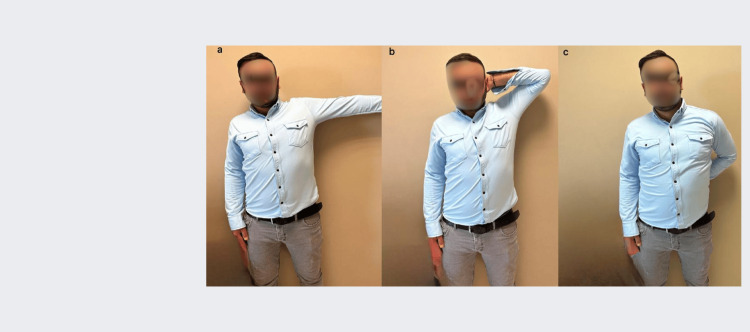
At the last follow-up of Patient 1, the movements of the shoulder in abduction (a), external rotation (b) and internal rotation (c) were not restricted and pain was not observed.

Postoperative antibiotic prophylaxis with first-generation cephalosporin was applied only for the first 24 hours. In the postoperative period, the patients were followed up with a bandage with the shoulder in a neutral position for one month (Figure 7). Only elbow and wrist movements were allowed for one month, while shoulder movements were completely restricted. After the end of the immobilization period, exercises including passive external rotation and forward flexion range of motion were started, supported by an acceptable active range of motion (ROM). Unrestricted activity and full ROM were allowed three months after surgery. CT scans of the shoulder were evaluated on postoperative day 1. Visual Analogue Scale (VAS) pain score, American Shoulder and Elbow Surgeons (ASES) shoulder score, and Constant-Murley Score (CMS) were used to evaluate functional outcomes. Shoulder ROM and possible complications were evaluated. 

Results

**Figure 8:**
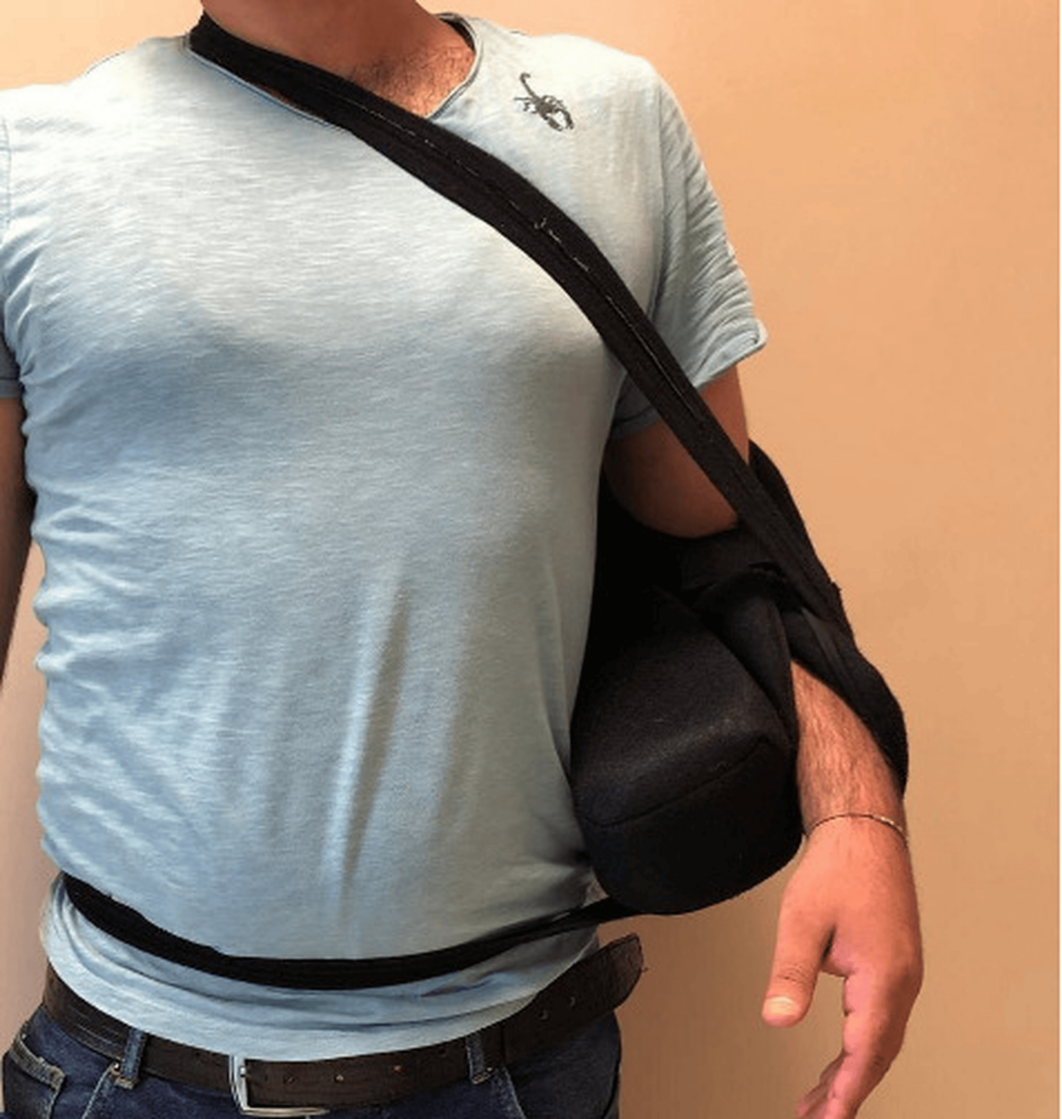
Immobilisation of the left shoulder in neutral position for one month postoperatively (Patient 1)

**Figure 9:**
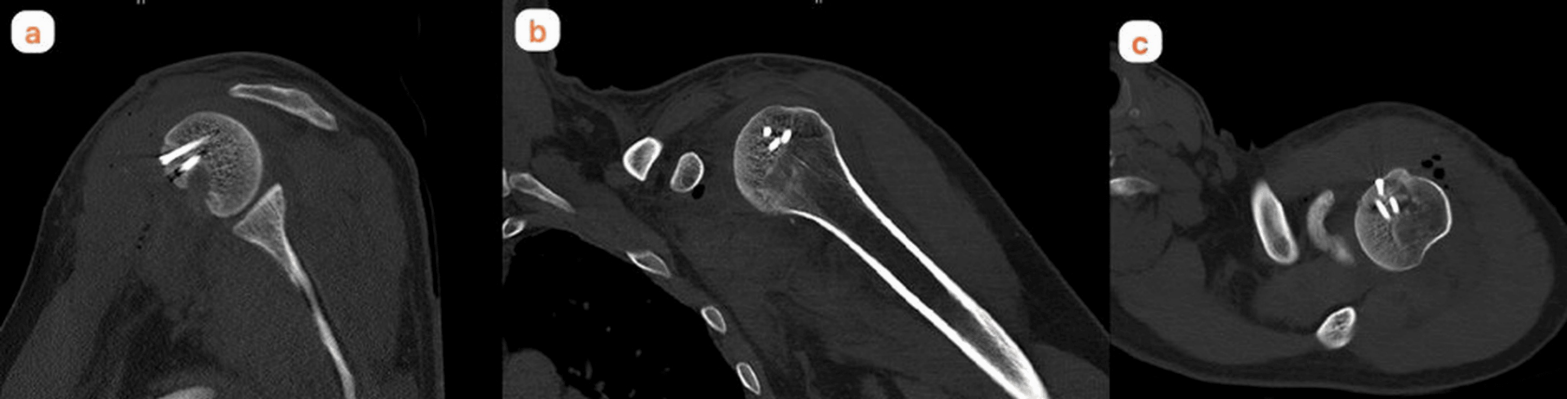
Sagittal (a), coronal (b) and axial (c) sections of the patient's left shoulder CT imaging at the last follow-up (Patient 1)

Patient 1 was followed up for 34 months and Patient 2 was followed up for 38 months. At the last follow-up, shoulder ROM in Patient 1 and Patient 2 was measured as forward flexion 170° and 175°, abduction 150° and 155°, internal rotation 60° and 65°, and external rotation 60° and 65°, respectively. There was no statistically significant difference in ROM measurements compared with the contralateral shoulder (p>0.05). At the last follow-up, the scores evaluating shoulder function for Patient 1 and Patient 2 were VAS 1 for each, ASES 90 and 96, respectively, and CMS 90 for each; there was no statistically significant difference between the scores of the patients (p>0.05). During the follow-up, shoulder instability was not observed in both patients and there was no pain and limitation of movement affecting daily life, and both patients were satisfied with the result (Figure 8). CT scans performed on postoperative day 1 and at the last follow-up showed that the articular surface restoration was anatomical and there were no problems with screw length and position (Figure 9). No signs of instability and arthrosis were observed in the three-year follow-up.

## Discussion

Locked posterior shoulder fracture-dislocation is a very rare injury. The gold standard treatment of this injury has not yet been clarified in the literature. We aimed to discuss the clinical and radiological results of two cases of locked posterior shoulder dislocation in which the impacted area of the humerus constituted 40% of the joint. We think that we obtained good results because the injuries of the patients in our study were treated with a modified Mclaughlin procedure in the acute phase, anatomical reduction was achieved on the articular surface and a meticulous surgical procedure was performed. In the opinion of the authors, posterior shoulder dislocations should not be overlooked and challenging maneuvers should be avoided in the emergency department to reduce posterior shoulder fracture dislocation. Early surgical operation and adherence to the determined techniques can achieve good results in the long term. The current study will contribute to the literature in terms of the limited number of patients treated in the acute period in the literature and the uncertainty in long-term results.

Locked posterior shoulder dislocation remains a confusing condition for orthopaedists due to its rarity, diagnostic difficulty, and lack of clarity in the current gold standard treatment [9]. It is estimated that approximately 60% of patients are misdiagnosed and dislocation is missed due to poor physical examination and inadequate radiographic imaging due to patient non-compliance and pain [10]. Clinical findings that may indicate the presence of posterior dislocation include palpable prominence of the coracoid, prominence of the humeral head in the posterior part of the shoulder, and restriction of external rotation, especially in the shoulder joint [11].

The development of reliable treatment guidelines has not yet been completed due to the small number of patients, the rarity of injuries, the paucity of clinical data and studies, and the ongoing controversy. While closed reduction and immobilization in external rotation are recommended for patients with reverse Hill-Sachs lesions with impaction of up to 25% of the humeral head and dislocations of less than three weeks duration, closed reduction is highly ineffective and may result in failure in patients with unreduced dislocations of more than three weeks duration and defects larger than 25% of the humeral head [12]. Surgical options for defects with 25-50% humeral head impaction include the McLaughlin procedure, modified McLaughlin procedure, rotational osteotomy, and autograft or allograft reconstruction [13-15].

Failure to treat posterior shoulder dislocation in the acute phase may result in permanent instability, osteonecrosis, chondrolysis, and consequent glenohumeral osteoarthritis [16].

The literature on the use of the modified McLaughlin procedure for posterior locked shoulder dislocation is still scant. Recent literature shows that this procedure has generally low complication rates, reduces the risk of recurrent instability, provides successful clinical and radiological results in long-term follow-up and that the modified McLaughlin procedure is a safe and effective treatment option in patients with a reverse Hill-Sachs lesion between 20% and 50% of the humeral head articular surface [17].

## Conclusions

This is one of the few studies sharing the results of using the modified McLaughlin procedure with an acute reduction in locked posterior fractured shoulder dislocations in which 40% of the articular cartilage is affected and closed reduction is not possible. Care should be taken not to overlook these rare injuries, and forceful reduction maneuvers should be avoided in the emergency department. With the modified Mclaughlin procedure, reduction and articular surface reconstruction can be performed in the same session and in the acute period. Acute treatment of the reverse Hill-Sachs lesion associated with locked posterior shoulder dislocation with the modified Mclaughlin procedure is effective and safe. Further studies are needed to determine the optimal treatment of these very rare injuries.
